# Age-Related Changes in the Perception of Emotions in Speech: Assessing Thresholds of Prosody and Semantics Recognition in Noise for Young and Older Adults

**DOI:** 10.3389/fnins.2022.846117

**Published:** 2022-04-25

**Authors:** Yehuda I. Dor, Daniel Algom, Vered Shakuf, Boaz M. Ben-David

**Affiliations:** ^1^School of Psychological Sciences, Tel Aviv University, Tel Aviv, Israel; ^2^Communication, Aging and Neuropsychology Lab (CANlab), Baruch Ivcher School of Psychology, Reichman University (IDC), Herzliya, Israel; ^3^Department of Communications Disorders, Achva Academic College, Arugot, Israel; ^4^Toronto Rehabilitation Institute, University Health Networks (UHN), Toronto, ON, Canada; ^5^Department of Speech-Language Pathology, University of Toronto, Toronto, ON, Canada

**Keywords:** auditory processing, speech perception, aging, semantics, emotions, noise, auditory sensory-cognitive interactions, prosody

## Abstract

Older adults process emotions in speech differently than do young adults. However, it is unclear whether these age-related changes impact all speech channels to the same extent, and whether they originate from a sensory or a cognitive source. The current study adopted a psychophysical approach to directly compare young and older adults’ sensory thresholds for emotion recognition in two channels of spoken-emotions: prosody (tone) and semantics (words). A total of 29 young adults and 26 older adults listened to 50 spoken sentences presenting different combinations of emotions across prosody and semantics. They were asked to recognize the prosodic or semantic emotion, in separate tasks. Sentences were presented on the background of speech-spectrum noise ranging from SNR of −15 dB (difficult) to +5 dB (easy). Individual recognition thresholds were calculated (by fitting psychometric functions) separately for prosodic and semantic recognition. Results indicated that: (1). recognition thresholds were better for young over older adults, suggesting an age-related general decrease across channels; (2). recognition thresholds were better for prosody over semantics, suggesting a prosodic advantage; (3). importantly, the prosodic advantage in thresholds did not differ between age groups (thus a sensory source for age-related differences in spoken-emotions processing was not supported); and (4). larger failures of selective attention were found for older adults than for young adults, indicating that older adults experienced larger difficulties in inhibiting irrelevant information. Taken together, results do not support a sole sensory source, but rather an interplay of cognitive and sensory sources for age-related differences in spoken-emotions processing.

## Introduction

Communication in older age is essential to maintain quality of life, cognitive skills, and emotional wellbeing (Heinrich et al., 2016; Livingston et al., 2017). Abundant evidence suggests that speech processing is impaired in aging, with severe implications (Helfer et al., 2017). Specifically, the literature points to major age-related changes in the perception of emotions in spoken language (Ben-David et al., 2019). However, it is not clear whether these changes are domain-specific or reflect a general age-related decline in emotion perception (Ruffman et al., 2008; Castro and Isaacowitz, 2019). In other words, do these changes stem from a specific deficit in processing of certain types of emotional channels (while processing of others is preserved), or from a general decrease in processing? In addition, there is debate on the mechanisms underlying these age-related changes; various sensory, cognitive, affective, and neural factors have been considered (Mather, 2016; Helfer et al., 2017; Ben-David et al., 2019).

In spoken language, emotions are presented via two main channels: (a) emotional semantics – the emotional meaning of spoken words or a complete sentence (segmental speech information); (b) emotional prosody – the tone of speech (suprasegmental speech information), composed of vocal cues such as stress, rhythm, and pitch. Processing of emotional speech is therefore a complex and dynamic integration of information, which may be congruent or incongruent, from these two channels. Significant age-related changes are indicated when incongruent prosody-semantics emotional combinations are presented. Specifically, when asked to integrate the two channels, young adults rely mainly on emotional prosody, while older adults weigh the two channels more equally (Dupuis and Pichora-Fuller, 2010; Ben-David et al., 2019). In addition, when listeners are asked to focus on only one speech channel, larger failures of selective attention are found for older adults than for young adults (Ben-David et al., 2019). In other words, the same spoken emotional sentences are interpreted differently by older and young listeners.

Mainly, cognitive and sensory sources have been suggested for these age-related differences (Ben-David et al., 2019). Following a cognitive source, age-related differences in executive functions, especially inhibition (Hasher and Zacks, 1988), are at the basis of changes in spoken emotion processing (Wingfield and Tun, 2001; Harel-Arbeli et al., 2021). Namely, both older and young adults may implicitly adopt the same weighting schematics – i.e., more weight to the prosodic than to the semantic channel. However, older adults might find it more difficult to inhibit the semantic information, processing it to a larger extent than intended.

An alternative sensory source lies in the relative imbalance between dimensions. The literature suggests that when one dimension becomes more perceptually salient than the other, the system is biased to rely on the first (Melara and Algom, 2003). Accordingly, young adults may be biased to process the prosody over the semantics, because emotional prosody is more sensory salient than emotional semantics. However, if this dimensional imbalance is reduced for older adults, the prosodic bias might be diminished as well (for a discussion on age-related sensory and dimensional-imbalance changes, see Ben-David and Schneider, 2009, 2010).

Some evidence in the literature may support this sensory source, with a specific age-related deficit in *prosodic processing* that might not be accompanied by a similar deficit in spoken-word processing. Indeed, age-related decrease in the recognition of prosodic information has been widely reported, both in quiet and in noise (Dmitrieva and Gelman, 2012; Lambrecht et al., 2012; Dupuis and Pichora-Fuller, 2014, 2015; Ben-David et al., 2019), suggesting a specific deficit in decoding emotional prosody in aging (Orbelo et al., 2005; Mitchell, 2007; Mitchell and Kingston, 2011). This prosodic deficit may relate to senescent changes in auditory brain areas and neural activity patterns (Orbelo et al., 2005; Giroud et al., 2019; Myers et al., 2019; Grandjean, 2021). However, there are mixed findings in the literature regarding the extent of age-related changes in *semantic processing*. While some studies have found a decline in older adults’ ability to extract the emotional meaning from words (Grunwald et al., 1999; Isaacowitz et al., 2007), other studies have maintained that semantic processing is preserved, at least when speech is presented in ideal listening conditions (Phillips et al., 2002; Ben-David et al., 2019). In sum, an age-related decrease in sensory dimensional imbalance may be the source for the age-related decrease in prosodic bias.

In the current study, we adopted a psychophysical approach to test the sensory base of age-related differences in processing of spoken emotions. Following the results obtained by Ben-David et al. (2019), we directly asked older and young listeners to recognize the prosodic emotion and semantic emotion of 50 spoken sentences in separate trials. Sentences were presented in five different signal-to-noise-ratios (SNRs) to calculate emotional recognition thresholds. Take, for example, the semantically happy sentence “I won the lottery” spoken with sad prosody. In previous studies, young adults were found to judge this sentence to convey mostly sadness (prosody), whereas older adults judged the sentence to present a similar extent of happiness (semantics) and sadness (prosody; Ben-David et al., 2019). A sensory source would be supported if a larger prosodic advantage in thresholds were to be found for young over older adults. A cognitive source would be supported if larger failures of selective attention were to be found for older adults, as gauged by accuracy differences between congruent and incongruent sentences. Note, the two sources are not mutually exclusive.

The following hypotheses were made:

1. *Age-related advantage*: Recognition thresholds and accuracy would be lower (i.e., better) for young than for older adults.
2. *Prosodic advantage*: Across age groups, recognition thresholds for emotional prosody would be lower (i.e., better) than for emotional semantics.
3. *Age-related differences in prosodic advantage*: As the literature is not clear, we did not wish to make an a-priori hypothesis as to whether the advantage in prosodic over semantic recognition thresholds would be affected by age group or not.
4. *Failures of selective attention*: Selective attention failures would be larger for older adults.

## Materials and Methods

### Participants

A total of 26 older adults from the community (16 women; 58-75 years old, *M* = 65.76 years, *SD* = 4.80) and 29 young adults, undergraduate students from Reichman University (24 women; 22-27 years old, *M* = 25.40 years, *SD* = 1.17) were recruited for this study and met the following inclusion criteria: (a) native Hebrew speakers as assessed by self-reports (Ben-David and Icht, 2018), and verified by above-average standard scores for their age range on a vocabulary test (subscale of the WAIS-III, Goodman, 2001), as language proficiency is related to processing of emotional semantics (Phillips et al., 2002); (b) good ocular health; no auditory, cognitive or language problems, and without any medical or mental conditions related to emotional processing as assessed by self-reports (Nitsan et al., 2019); (c) no indication of clinical depression as assessed by self-reports (older: GDS, Zalsman et al., 1998; young: DASS-21, Henry and Crawford, 2005); and (d) pure-tone air-conduction thresholds within clinically normal limits for their age group, for 500, 1,000, and 2,000 Hz (average pure-tone thresholds ≤ 15 dB HL for young, and ≤ 25 dB HL for older adults, difference between ears < 20 dB HL). Note, groups were matched on years of education (*M* = 14.23 and 14.19 for young and older adults, respectively), taken as a reliable gauge for linguistic skills (Kaufman et al., 1989; Ben-David et al., 2015). Young adults participated in the study for partial course credit, and older adults were compensated by the equivalent of $10. From the final dataset, we excluded data of two young participants who did not follow the instructions, and of four older adults who exhibited very low recognition rates (< 50% correct recognition in the easiest SNR). A detailed description of the demographic and audiological characteristics of participants can be found in Supplementary Appendix A.

### Stimuli

**FIGURE 1:**
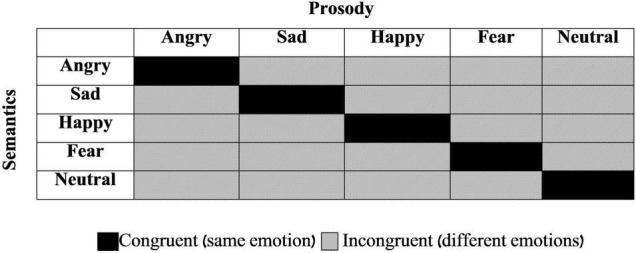
General design of the Stimuli. All combinations of prosodic and semantic emotions are presented. Each cell represents two different sentences used in this study. Black cells: congruent sentences (same emotion in both speech channels). Gray cells: incongruent sentences (different emotions in semantics and prosody).

The stimulus set was made of 50 spoken sentences taken from the Test for Rating of Emotions in Speech (T-RES; Ben-David et al., 2016, 2019), which presents emotional semantic and prosodic content in different combinations from trial to trial. Five different emotions were used: Anger, Happiness, Sadness, Fear, and Neutrality. Each semantic category was represented in each of the tested prosodies, generating a 5 (semantics) *5 (prosody) matrix (see Figure 1). The experimental set consisted of two sentences in each of the 25 different combinations of emotional semantics and prosody. Ten sentences were congruent (e.g., semantically angry semantics such as “Get out of my room” spoken with congruent angry prosody; black cells in Figure 1) and 40 were incongruent (e.g., semantically happy semantics such as “I won the lottery” spoken with incongruent sad prosody; gray cells in Figure 1). All spoken sentences were recorded by a professional radio drama actress; digital audio files were equated with respect to their duration and root-mean-square amplitude (before they were mixed with noise).

### Reliability, Sensitivity, and Validity

We used the Hebrew version of the T-RES sentences. Content validity (Chan, 2014) was confirmed by verifying that all semantic stimuli were distinctive in their categories and exemplars of their respective semantic categories for both young and older listeners, and equated on main linguistic characteristics. For full details on the procedure for stimuli selection, see Ben-David et al. (2011b,^2013^, 2019). A recent study from our lab has further shown that the discrete prosodic emotions are clearly distinct in acoustic characteristics (mean F0 and speech rate; Carl et al., 2022) in this set. The T-RES reliability was confirmed as data for young adult undergraduates were found to be equivalent across studies and platforms (Ben-David et al., 2021). The T-RES stimuli were also found to be valid and sensitive in detecting population-related differences in various studies. For example, expected differences in spoken emotional processing were found when comparing cochlear implant users and their peers (Taitelbaum-Swead et al., 2022).

### Sentence Division and Combination With Noise

The final set was divided into five subsets of ten sentences each, with each subset consisting of two congruent and eight incongruent sentences. Each of the five emotional prosodies and each of the five emotional semantic categories was represented twice in each subset (see Supplementary Appendix B). Using PRAAT software (Boersma and Weenink, 2019), stimuli in each subset were combined with a different level of background speech-spectrum noise using a standard steady-state noise masker taken from the Revised Speech Perception in Noise test (Bilger et al., 1984; for spectral analysis of this noise, see Figure 6 in Ben-David et al., 2012). Five SNR levels were used: −15 dB, −10 dB, −5 dB, 0 dB, and +5 dB; creating a scale from the most difficult SNR (−15 dB) to the easiest SNR (+5 dB).

### Procedure and Apparatus

Upon arrival, all participants received a short explanation regarding the experimental task and signed an informed consent form. Participants completed the self-reports and the vocabulary test. Next, they were seated in an IAC sound-attenuated booth and performed the pure-tone hearing thresholds test. All auditory stimuli were presented via MAC-51 audiometer headphones. Spoken sentences (experimental task) were presented 40 dB above individual audiometric thresholds (pure-tone average) in quiet, to partially mitigate age-related differences in auditory thresholds. Instructions were presented on a 17-in. flat color monitor.

### Experimental Session

The experimental session consisted of two five-alternative-forced-choice (5-AFC) tasks. In both, participants were instructed to recognize the emotion presented, choosing one of five options (anger, happiness, sadness, fear, and neutrality) by pressing a designated key on the keyboard. Listeners were asked to recognize only the emotion presented by the semantics in the Semantics-recognition task, or only by prosodics in the Prosody-recognition task. Each task consisted of five blocks of ten spoken sentences each, with different levels of SNR in each block. The order of tasks (Semantics-recognition or Prosody-recognition) and the order of blocks in each task were counterbalanced across participants, using a Latin-square design. The order of sentences within each block was fully randomized. The whole session (two tasks with 100 sentences in total) lasted less than 30 min. Participants were given the option to take short breaks before the session, or between the tasks, if needed.

### Data Fitting and Psychometric Functions

For each participant, five recognition-accuracy rates were calculated separately for prosody and semantics, based on average accuracy across the ten sentences in each of the five SNRs. Using a customized MATLAB script (McMurray, 2017), data were fitted to the logistic psychometric function of the form,


(1) $$f\left(x\right)=A+\frac{L-A}{1+e^{-k\left(x-x_{0}\right)}},$$

where *f(x)* represents recognition-accuracy rates, *x* is the SNR in dB, *L* and *A* are the upper and lower asymptotes of the function, respectively. Most importantly, the parameter *x*_0_ represents the function’s crossover point, or the *x* value that corresponds to middle performance between the boundaries of the function. The crossover point is taken to represent the point at which the rate of increase in recognition as a function of SNR begins to decrease. As such, the value of *x*_0_ can serve as an index for individual recognition statistical threshold (Ben-David et al., 2012; Morgan, 2021). Finally, *k* represents the function’s slope at *x*_0_.

The lower asymptote of the function (*A*) for all conditions was pre-defined as 0.2 (chance level) using two techniques: (1) All performance levels averaging under 0.2 were corrected to 0.2 to avoid function estimations below chance level (1.4% of the data corrected). (2) We added an estimation level of 0.2 recognition rates (chance level) for an SNR of −20 dB, to correspond to the function’s predicted lower bound. However, we chose not to pre-define the upper bound of the function (i.e., maximum recognition rates, see Morgan, 2021), as even without any background noise emotional recognition rates are not expected to reach 100%, especially for older adults (see Ruffman et al., 2008; Ben-David et al., 2019). Hence, the three other parameters (*x_0_, L*, and *k*) were estimated based on our data. Correlations between actual data and the values predicted by the psychometric function were high (Mean correlation, 0.98–0.95), indicating a very good fit (McMurray, 2017) for both young and older adults. For full details regarding recognition rates, fitted psychometric functions’ parameters, and quality of fits, see Supplementary Appendices C and D.

### Statistical Analysis

All analyses of the thresholds, maximum asymptotes, and slopes (*x*_0_, *L*, and *k*, taken from the psychometric function) included mixed linear modeling, MLM (SPSS Statistics 20; IBM Corp, 2011), with each serving as the dependent variable in different models. Group (young adults vs. older adults) was the between participant variable and Speech Channel (Prosody-rating vs. Semantics-rating) was the within participant variable. To test Selective Attention, the same MLM model was used, with recognition-accuracy rates (averaged across all SNRs) as the dependent variable, and the Selective Attention factor (congruent vs. incongruent sentences) added as another within participant factor.

## Results

### Analysis of Thresholds and Recognition Rates

Table 1A presents the full MLM analyses of recognition thresholds, maximum asymptotes, and slopes. Results indicated a significant main effect for Age Group, *F*(1,47) = 14.57, *p* < 0.001, suggesting lower recognition thresholds for young, compared to older adults (average thresholds of −9.57 dB vs. −7.37 dB, respectively). A significant main effect was also found for Speech Channel, *F*(1,47) = 74.98, *p* < 0.001, suggesting lower recognition thresholds for emotions in prosody, compared to semantics (average thresholds of −10.09 dB vs. −6.85 dB, respectively). However, the interaction of the two factors was not significant, *F*(1,47) = 1.24, *p* = 0.27, indicating that the prosodic threshold advantage was similar for both age groups (left column of Table 1A). When using the same model to test differences in maximum asymptotes (i.e., maximal recognition rates under minimal noise) significant main effects were found for Age Group, *F*(1,47) = 27.62, *p* < 0.001, and for Speech Channel, *F*(1,47) = 5.34, *p* = 0.025, without a significant interaction between the two, *F*(1,47) = 0.304, *p* = 0.584 (middle column of Table 1A). When the same model was used to test differences in slopes, none of the tested effects were significant, indicating similar growth rates across all conditions (right column of Table 1A). When we excluded from analysis all psychometric functions whose fit quality was less than 0.9 (excluding seven functions, 7% of data), the result pattern remained the same (see Supplementary Appendix E).

**TABLE 1:** Model Summary and results of MLM analyses.

A: Psychometric Function’s Parameters			
	Threshold	Max recognition	Slope
Age Group	*F*(1,47) = 14.57, *p* < 0.001	*F*(1,47) = 27.62, *p* < 0.001	*F*(1,47) = 1.17, *p* = 0.285
Speech Channel	*F*(1,47) = 74.98, *p* < 0.001	*F*(1,47) = 5.34, *p* = 0.025	*F*(1,47) = 1.04, *p* = 0.313
Age Group X Speech Channel	*F*(1,47) = 1.24, *p* = 0.272	*F*(1,47) = 0.304, *p* = 0.584	*F*(1,47) = 0.233, *p* = 0.632
Model Summary	BIC = 448.87	BIC = −135.16	BIC = −53.79

**B: Selective Attention**			

			**Recognition rates**

Age Group			*F*(1,42.82) = 20.45, *p* < 0.001
Speech Channel			*F*(1,40.64) = 12.88, *p* = 0.001
Selective Attention			*F*(1,44.04) = 55.6, *p* < 0.001
Age Group X Speech Channel			*F*(1,40.64) = 0.809, *p* = 0.374
Age Group X Selective Attention			*F*(1,44.04) = 10.2, *p* = 0.003
Speech Channel X Selective Attention			*F*(1,32.13) = 2.76, *p* = 0.106
Age Group X Speech Channel X Selective Attention			*F*(1,32.13) = 3.1, *p* = 0.088
Model Summary			BIC = −161.72

*Top panel: analysis of individual psychometric functions’ parameters (left column: Thresholds, x_0_ parameter; middle column: Max recognition, L parameter, maximum asymptote; right column: Slope, k parameter) for all data. Bottom panel: analysis of Selective Attention effects (difference between recognition rates of emotions in congruent and incongruent sentences). Significant effects are shaded.*

**FIGURE 2:**
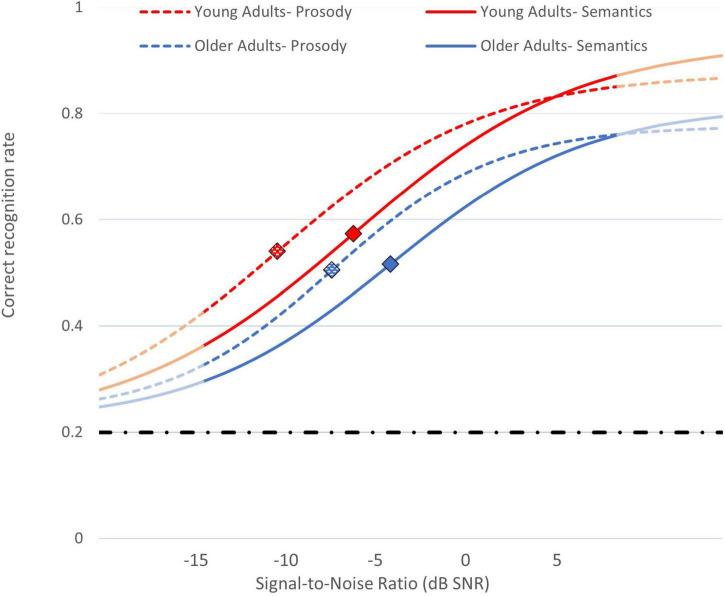
Psychometric functions for recognition of emotions in speech in different SNRs, averaged across participants. Blue lines: older adults; red lines: young adults. Dashed lines: recognition of emotional prosody; full lines: recognition of emotional semantics. Diamond-shaped markers indicate statistical recognition thresholds for each condition. Light-blue and light-red lines represent extrapolations of the functions beyond the SNRs tested in the study. The dashed-and-dot horizontal line indicates the functions’ minimal asymptote (0.2 - chance level).

To sum, our first and second hypotheses were confirmed: Young adults’ recognition thresholds were lower (better) than those of older adults (a difference of about 2.2 dB), and prosodic emotions yielded lower recognition thresholds than did semantics emotions (a difference of about 3.3 dB). Critically, regarding our third hypothesis, the relative extent of the advantage of prosody over semantics was highly similar for older and young adults. Namely, prosodic thresholds were better than semantic thresholds by about a third, 32.16%, and 32.06% (−8.98 vs. −5.96 dB SNR; and, −11.40 vs. −7.74 dB SNR) for older and young adults, respectively. These results and the estimated psychometric functions in different Age Groups and Speech Channels are visually presented in Figure 2.

### Analysis of Selective Attention Failures

Table 1B presents the full MLM analyses of the Selective Attention factor. Results show significant main effect for Age Group, *F*(1,42.82) = 20.45, *p* < 0.001, and for Speech Channel, *F*(1,40.64) = 12.88, *p* = 0.001, with no significant interaction between the two, *F*(1,40.64) = 0.809, *p* = 0.374, conceptually replicating the results reported above. Most importantly, we found a significant main effect for Selective Attention, *F*(1,44.04) = 55.6, *p* < 0.001, that significantly interacted with Age Group, *F*(1,44.04) = 10.2, *p* = 0.003, reflecting larger failures of selective attention for older adults.

**FIGURE 3:**
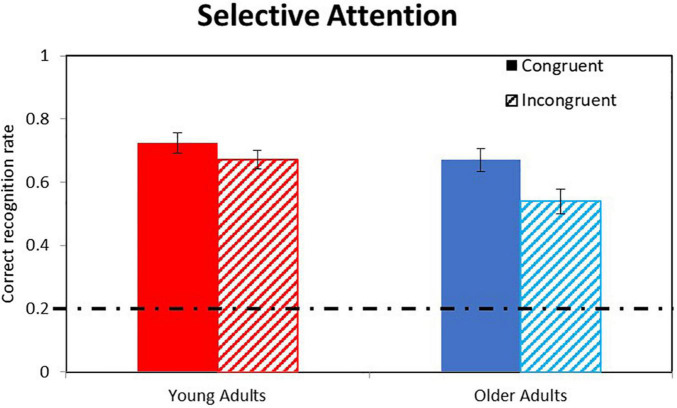
Analysis of Selective Attention effects. Bars indicate correct recognition rates for congruent (full) vs. incongruent (dashed) sentences in young (red) and older (blue) adults averaged across different SNRs. Error bars indicate 95% CI of their respective means (MLM estimates). The dashed-and-dot horizontal line indicates the chance level (0.2).

To sum, our fourth hypothesis was confirmed: Recognition rates were better for congruent than for incongruent sentences (correct recognition rates of 0.697 vs. 0.606, respectively), indicating overall failures of selective attention. Older adults showed larger failures of selective attention than did young adults (Selective-Attention factors of 0.130 vs. 0.052, respectively). These results are visually presented in Figure 3.

## Discussion

The current study adopted a psychophysical approach to directly compare young and older adults’ sensory thresholds for emotion recognition across two channels of speech: prosody and semantics. We aimed to better understand age-related differences in the processing of spoken emotions, as indicated in the literature, and specifically an age-related decrease in the dominance of prosody over semantics, as found by Ben-David et al. (2019). A total of 29 young adults and 26 older adults listened to 50 spoken sentences presenting different combinations of emotions across prosody and semantics and, in different tasks, were asked to recognize the emotion presented in one of the channels. Sentences were mixed with speech-spectrum noise ranging from SNR of −15 dB (most difficult) to +5 dB (easiest). Individual recognition thresholds were calculated (by fitting psychometric functions), separately for prosodic and for semantic emotion recognition.

Results indicated the following trends, supporting our hypotheses:

1. Recognition thresholds were better for young over older adults (*age-related effects*);
2. Recognition thresholds were better for prosodic over semantic information (*prosodic advantage*);
3. The prosodic advantage in thresholds did not differ between age-groups;
4. However, a significant age-related effect was indicated for *selective attention*, suggesting that older adults were more affected by the irrelevant channel than were young adults.

To the best of our knowledge, this study is the first to directly examine possible age-related differences in the imbalance between thresholds for emotion recognition in different speech channels. To date, only a few studies have tried to directly compare the recognition of the semantic and prosodic channels, mostly in young adults (but see, Dupuis and Pichora-Fuller, 2014). For example, Morgan (2021) showed that sensory thresholds were better for prosodic-emotion recognition than for word recognition in noise for young adults (see also van Zyl and Hanekom, 2011; Ritter and Vongpaisal, 2018; Morgan et al., 2022). However, these studies did not directly measure semantic-emotion recognition, but rather used word/sentence recognition as a placeholder. Clearly, these two processes differ, as semantic-emotion recognition involves both the identification of the spoken words and their integration as the basis for emotional labeling.

As expected, we found lower (better) recognition thresholds for young over older adults. In other words, older adults needed speech to be presented at ∼2.2 dB SNR louder than young adults to reach their recognition threshold in noise. These results are in line with the abundant literature on speech perception in noise (Heinrich et al., 2016). *Semantics:* Age-related changes in semantic emotion recognition follow findings on spoken word recognition. Note, our effects are about half the size of the well-observed 4 dB SNR age-related difference in spoken-word recognition accuracy (Pichora-Fuller et al., 1995; Murphy et al., 2000; Ben-David et al., 2011a). This is probably the outcome of the different tasks used, as we tested emotion recognition thresholds rather than word recognition accuracy. *Prosody:* The current study is the first to directly test age-related changes in recognition thresholds for emotional prosody. Our findings, on an age-related decrease in prosodic recognition thresholds, expand previous findings on age-related diminished prosodic recognition accuracy for speech in noise (Dmitrieva and Gelman, 2012; Dupuis and Pichora-Fuller, 2014). *Maximum asymptotes:* An age-related difference was found for the maximum asymptote of the psychometric functions, indicating that young adults recognize emotions in speech better than older adults, even under very little noise (see Paulmann et al., 2008; Ben-David et al., 2019). Recognition accuracy for older adults did not reach 100% at the maximum asymptotes (easiest SNR). This is not surprising, as the literature suggests that even in quiet older adults are impaired at emotion recognition (Ruffman et al., 2008), speech recognition (Pichora-Fuller and Souza, 2003) and emotional prosody and semantics recognition (Paulmann et al., 2008).

Our results support a sensory prosodic advantage across both age-groups, where recognition thresholds were lower (better) for emotional prosody than for emotional semantics. This suggests that to reach recognition threshold in noise, emotional semantics call for an addition of ∼3.3 dB SNR as compared to emotional prosody. This prosodic advantage across age groups expands previous evidence that focused mainly on an accuracy advantage for prosodic recognition over spoken word recognition (Dupuis and Pichora-Fuller, 2014; Morgan, 2021; Morgan et al., 2022). A noteworthy study by Morgan (2021) reported a 10 dB SNR advantage between emotional prosodic thresholds and spoken word identification thresholds in young adults. This marks a much larger advantage than the 3.3 dB SNR difference we report. This difference possibly stems from the tasks used (word identification vs. emotion recognition in a sentence) and from other methodological differences (such as the different levels of SNRs used in each condition). *Maximum asymptotes:* In contrast to the prosodic advantage in SNR thresholds, it is notable that a small but significant semantic advantage was found for the maximum asymptote of the psychometric functions, indicating that emotional semantics are recognized slightly better than are emotional prosodies under very little noise (see also Ben-David et al., 2019).

How to explain this ease of prosodic detection in noise? As aforementioned, spoken emotional semantic recognition is based on both word identification and context generation as the words unfold in time. These tasks are highly sensitive to noise (Pichora-Fuller et al., 1995), as misapprehension of sound-sharing words might change the emotional meaning of the whole sentence. For example, consider the sentences “I’m so */sad/* right now” versus “I’m so */mad/* right now.” Confusing one phoneme for another, a common characteristic of speech-in-noise processing (Ben-David et al., 2011a; Nitsan et al., 2019), shifts the emotional categorization of the sentence from sadness to anger. In contrast, prosodic recognition is based on suprasegmental features that may be less susceptible to noise. Namely, prosodic processing is based on the envelope of speech, speech rate and fundamental frequency fluctuations (Myers et al., 2019). These acoustic features are more immune to interference from energetic masking (Morgan, 2021). Moreover, processing of prosodic features involves several functionally (and anatomically) segregated systems of cortical and sub-cortical networks (Grandjean, 2021). This redundancy might serve to protect from the effects of adverse sensory conditions.

Indeed, prosody has been taken to be a fundamental aspect of speech that scaffolds other aspects of linguistic processing (Myers et al., 2019). Emotional prosody is learned and used already in infancy, before the effective use of semantics in infant-parent interactions (Fernald, 1989). Thus, prosody serves as a basic emotional cue across the life span. Prosody also appears to be a contextualizing marker of verbal interactions that directly leads listeners to the speaker’s emotional message (House, 2007). The critical role prosody plays in interpersonal and social situations (Pell and Kotz, 2021) may be generated by its perceptual salience, or may lead to heightened sensitivity to prosodic cues in noise.

Perhaps our most important finding is the lack of interaction between age group and prosodic advantage in sensory thresholds. In other words, the prosodic advantage was similar in extent for older and young adults (around a 33% advantage in both groups). Our data do not support suggestions in the literature that older adults might have specific impairments in prosodic processing as compared to young adults (Mitchell, 2007; Orbelo et al., 2005). Rather, they are in line with a general age-related auditory decline that spans to both segmental and suprasegmental features (Paulmann et al., 2008). Results could also support a general age-related decrease in emotional perception and processing (Ruffman et al., 2008; but see Castro and Isaacowitz, 2019) across the two speech channels.

In contrast to the preserved prosodic advantage in recognition thresholds, we observed significant age-related differences in selective attention. When asked to focus on one speech channel, older adults were affected to a larger extent by the content of the other, irrelevant channel. This finding could be taken to support the age-related inhibitory deficit hypothesis (Hasher and Zacks, 1988; Ben-David et al., 2014), with older adults experiencing larger difficulties in inhibiting irrelevant information. Alternatively, our results could be based on an information degradation hypothesis (Schneider and Pichora-Fuller, 2000; Ben-David and Schneider, 2009, 2010), whereby age-related sensory changes lead to performance changes. In the current study, information in the prosodic and semantic channels was degraded to a similar extent due to auditory sensory degradation in aging. Clearly, pure-tone thresholds for older adults were significantly worse than for young adults (see Supplementary Appendix A). These and other age-related audiological changes (e.g., frequency selectivity and loudness recruitment; Füllgrabe, 2020) are likely to have had an impact on age-related sensory degradation of speech perception. Consequently, older adults in our study might have adopted a wider processing strategy and integrated information from both speech channels to form a clearer picture of the speaker’s intent (Hess, 2005, 2006, 2014). Whereas this strategy improves processing in congruent prosody-semantic sentences, it leads to failures in selective attention in incongruent sentences.

It is notable that older adults in our sample experienced a larger extent of hearing loss in the higher frequencies (4,000 and 8,000 Hz, see Supplementary Appendix A). This high-frequency hearing loss is common for older adults with clinically normal hearing (in the lower frequency ranges) recruited for speech processing studies (Dupuis and Pichora-Fuller, 2014, 2015; Nagar et al., 2022). It has been suggested that this age-related difference may have a specific effect on semantic processing, as many speech cues are available in a range around 4,000 Hz (Vinay and Moore, 2010); whereas prosodic cues, such as f0 and the envelope of speech, might still be preserved. Our findings do not necessarily support this option, as we found an equivalent SNR prosodic advantage for older and young adults. In other words, age-related sensory degradation appears to have had a similar impact on semantic and prosodic emotional processing in the current study. Thus, our results follow the literature indicating that age-related sensory changes are not the sole source of difficulties older adults experience when speech is presented in adverse listening conditions (Roberts and Allen, 2016). For example, Füllgrabe et al. (2015) found age-related deficits in speech-in-noise identification to persist even when audiograms for older and young adults were matched (see also Grassi and Borella, 2013). Following Cardin (2016), listening in adverse conditions becomes effortful in aging and demands more cognitive resources, thus speech processing is affected by age-related changes in both sensory and cognitive factors.

### Caveats, Future Directions, and Implications

Limitations of the current study include relatively small numbers of participants in each age group. However, this number is not different than that found in the pertinent literature (e.g., 20 participants, Morgan, 2021). Even though the range of SNR used was large enough to include individual thresholds, future studies may increase the range to improve the assessment’s accuracy. In addition, the current study used speech-spectrum noise, a standard noise type widely used in age-related comparisons (Ben-David et al., 2011a,^2012^). Future studies may wish to test further types of auditory distortions (e.g., Ritter and Vongpaisal, 2018; Dor et al., 2020). Future studies may also test the effects of individual audiometric thresholds (see Grassi and Borella, 2013), demographic characteristics (e.g., gender, socio-economic status and education), as well as emotional traits and mental health (e.g., empathy and alexithymia, see Leshem et al., 2019) on emotion recognition thresholds. Indeed, mental health was also found to affect the recognition of negative and positive emotions differently (e.g., detection of emotionally negative words was related to PTSD and forensic schizophrenia; Cisler et al., 2011; Leshem et al., 2020). Finally, this study used a unique set of validated and standardized spoken sentences that present emotional content in both semantics and prosody. Future studies may wish to expand the scope of this study’s findings by using different sets of sentences.

In sum, the current study is the first to directly compare emotion recognition thresholds for spoken semantics and prosody in young and older adults. Mainly, we found a recognition threshold advantage for young over older adults, an advantage for prosody over semantics that was not affected by age group, and larger failures of selective attention for older adults. Previous studies indicate that older adults assign different relative weights to prosodic and semantic spoken emotions than do young adults, possibly resulting in an inter-generational communication breakdown (Dupuis and Pichora-Fuller, 2010; Ben-David et al., 2019). The current study does not support a sensory source for this age-related difference in speech processing, hinting to a possible cognitive source. Future studies should directly test whether processing of prosodic and semantic emotions demands a different extent of cognitive resources for young and older adults.

## Data Availability Statement

The raw data supporting the conclusions of this article will be made available by the authors, without undue reservation.

## Ethics Statement

The studies involving human participants were reviewed and approved by Ethics Committee of Reichamn University, Herzliya, Israel. The patients/participants provided their written informed consent to participate in this study.

## Author Contributions

YD and BB-D wrote the manuscript, they are responsible for the design of the paradigm, the analysis and interpretation of the data. DA and BB-D supervised the research project, DA and VS made invaluable contributions to the conceptualizing the research question and the final manuscript. BB-D is the corresponding author and the study was conducted in his lab. All authors had a prominent intellectual contribution to the study, are accountable for the data and approved the final version of the manuscript.

## Conflict of Interest

The authors declare that the research was conducted in the absence of any commercial or financial relationships that could be construed as a potential conflict of interest.

## Publisher’s Note

All claims expressed in this article are solely those of the authors and do not necessarily represent those of their affiliated organizations, or those of the publisher, the editors and the reviewers. Any product that may be evaluated in this article, or claim that may be made by its manufacturer, is not guaranteed or endorsed by the publisher.
