# Supplementary material for: Age-Related Changes in the Perception of Emotions in Speech: Assessing Thresholds of Prosody and Semantics Recognition in Noise for Young and Older Adults
Source: Front Neurosci. 2022 Apr 25;16:846117. doi: 10.3389/fnins.2022.846117 (PMC9082150; doi:10.3389/fnins.2022.846117)
Supplement: Supplementary file 1 [file Table_1.pdf]

frontiers

Supplementary Material

Age-related changes in the perception of emotions in speech: assessing thresholds of prosody and semantics recognition in noise for young and older adults

Appendix A.

		Young Adults	Older adults
**Demographic variables**	**Age***	*M*=25.40 years, *SD*=1.17	*M*=65.76 years, *SD* = 4.80
	**Gender**	24 women, 5 men	16 women, 10 men
	**Years of education**	*M*=14.23 years, *SD* = 1.31	*M*=14.19 years, *SD*=3.31
	**Income (self-reported on a 5-point Likert scale with 3=average income) ***	*M*=2.57, *SD* = 1.53	*M*=4.11, *SD*=.88
**Pure-tone air-conduction thresholds**	**250 Hz***	*M*=9.63 dB, *SD* = 4.48	*M*=16.04 dB, *SD*=4.45
	**500 Hz***	*M*=8.24 dB, *SD* = 3.85	*M*=17.71 dB, *SD*=4.05
	**1000 Hz***	*M*=7.96 dB, *SD* = 4.33	*M*=17.08 dB, *SD*=6.20
	**2000 Hz***	*M*=7.96 dB, *SD* = 5.46	*M*=23.18 dB, *SD*=10.55
	**4000 Hz***	*M*=7.68 dB, *SD* = 4.80	*M*=27.86 dB, *SD*=9.18
	**8000 Hz***	*M*=7.50 dB, *SD* = 4.44	*M*=27.14 dB, *SD*=13.49
**Pure-tone average threshold**	**Average of 500, 1000 & 2000 Hz***	*M*=8.06 dB, *SD* = 3.24	*M*=19.08 dB, *SD* = 4.74

Appendix A. Demographic and audiological characteristics of participants in the study. Variables with significant difference between age groups of p<.001 (using χ^2 test for gender and independent samples t-test for all other variables) are marked with asterisks. Unmarked variables were not significantly different between groups (P>.05).

Appendix B.

Subset (block) 1: SNR=-15 dB

Semantics \ Prosody	Angry	Sad	Happy	Fear	Neutral
Angry		*		*	
Sad		*			*
Happy			*		*
Fear	*		*		
Neutral	*			*	

Subset (block) 2: SNR=-10 dB

Semantics \ Prosody	Angry	Sad	Happy	Fear	Neutral
Angry	*				*
Sad			*	*	
Happy	*	*			
Fear				*	*
Neutral		*	*		

Subset (block) 3: SNR=-5 dB

Semantics \ Prosody	Angry	Sad	Happy	Fear	Neutral
Angry			*		*
Sad	*		*		
Happy	*			*	
Fear		*		*	
Neutral		*			*

Subset (block) 4: SNR=0 dB

Prosody

Semantics	Angry	Sad	Happy	Fear	Neutral
**Angry**		*		*	
**Sad**		*			*
**Happy**			*		*
**Fear**	*		*		
**Neutral**	*			*	

Subset (block) 5: SNR=+5 dB

Prosody

Semantics	Angry	Sad	Happy	Fear	Neutral
**Angry**	*		*		
**Sad**	*			*	
**Happy**		*		*	
**Fear**		*			*
**Neutral**			*		*

Appendix B. Division of the 50 sentences in the dataset into five subsets (blocks) of ten sentences each. Each subset consisted of two congruent and eight incongruent sentences. Each of the five emotional prosodies and each of the five emotional semantic categories was represented twice in each subset of sentences. Each subset was mixed with a different level of background speech-spectrum noise ranging from SNR=-15 dB to SNR=+5 dB. Asterisks represent the emotional prosody and semantics of sentences included in each subset.

Appendix C.

			Recognition Rates					Fitted Parameters			
**Participant No.**	**Age Group**	**Speech Channel**	**SNR= -15 dB**	**SNR= -10 dB**	**SNR= -5 dB**	**SNR= 0 dB**	**SNR= +5 dB**	***L* (max asymptote)**	**x_0 (threshold)**	***k* (slope)**	**r (quality of fit)**
101	YA	Prosody	0.3	0.7	0.7	0.6	1	0.769	-12.761	0.097	0.894
101	YA	Semantics	0.2	0.2	0.5	0.8	0.9	0.889	-4.327	0.089	0.998
102	YA	Prosody	0.1	0.8	0.8	1	1	0.934	-11.212	0.226	0.981
102	YA	Semantics	0.2	0.3	0.7	1	1	1.000	-6.050	0.107	0.999
103	YA	Prosody	0.2	0.7	0.9	0.7	1	0.867	-10.382	0.480	0.960
103	YA	Semantics	0.4	0.6	0.8	0.9	1	0.992	-9.855	0.049	0.994
104	YA	Prosody	0.3	0.6	0.8	0.9	1	0.970	-9.660	0.060	0.995
104	YA	Semantics	0.3	0.4	0.9	1	1	1.000	-7.937	0.119	0.997
105	YA	Prosody	0.3	0.4	0.8	0.9	0.9	0.900	-8.058	0.090	0.996
105	YA	Semantics	0.2	0.3	0.8	0.9	0.8	0.850	-7.984	0.138	0.995
106	YA	Prosody	0.2	0.3	0.6	0.8	0.5	0.646	-8.269	0.082	0.919
106	YA	Semantics	0.2	0.4	0.8	0.9	1	0.965	-7.687	0.088	0.997
107	YA	Prosody	0.4	0.6	0.7	0.9	0.8	0.843	-11.491	0.046	0.979
107	YA	Semantics	0.2	0.4	0.5	0.9	1	1.000	-4.337	0.069	0.987
108	YA	Prosody	0.5	0.7	0.6	0.9	0.8	0.773	-14.718	0.061	0.921
108	YA	Semantics	0.1	0.6	0.5	0.9	0.8	0.863	-7.983	0.042	0.917
109	YA	Prosody	0.5	0.6	0.7	0.8	0.8	0.790	-13.384	0.041	0.975
109	YA	Semantics	0.2	0.7	0.9	1	0.9	0.934	-10.702	0.197	0.995
110	YA	Prosody	0.6	1	0.9	0.8	1	0.925	-15.078	0.480	0.971
110	YA	Semantics	0.4	0.5	1	0.9	1	0.967	-9.706	0.480	0.977
111	YA	Prosody	0.3	0.7	0.8	0.7	1	0.839	-12.134	0.091	0.950
111	YA	Semantics	0.4	0.6	0.7	0.9	1	1.000	-8.945	0.043	0.987
112	YA	Prosody	0.2	0.5	0.9	0.8	0.9	0.867	-9.920	0.420	0.994
112	YA	Semantics	0.3	0.4	0.8	1	1	1.000	-7.313	0.088	0.997
113	YA	Prosody	0.4	0.6	0.9	1	0.9	0.965	-10.906	0.065	0.988
113	YA	Semantics	0.2	0.6	0.6	1	1	1.000	-7.500	0.056	0.960
114	YA	Prosody	0.4	0.7	0.7	0.9	0.9	0.876	-12.134	0.052	0.976
114	YA	Semantics	0.2	0.6	0.5	1	0.9	1.000	-6.441	0.049	0.923
115	YA	Prosody	0.5	0.9	0.9	0.8	1	0.900	-14.871	0.391	0.978
115	YA	Semantics	0.3	0.5	0.8	0.9	1	0.990	-8.455	0.060	0.998
116	YA	Prosody	0.2	0.6	0.9	0.8	1	0.900	-10.117	0.430	0.983
116	YA	Semantics	0.2	0.6	0.7	0.9	1	0.986	-8.195	0.056	0.979

117	YA	Prosody	0.2	0.3	0.5	0.9	0.7	0.800	-4.960	0.420	0.967
117	YA	Semantics	0.2	0.1	0.7	0.9	0.7	0.800	-5.576	0.419	0.980
118	YA	Prosody	0.2	0.6	0.9	0.8	1	0.900	-10.117	0.431	0.983
118	YA	Semantics	0.2	0.2	0.6	0.9	0.9	0.900	-5.120	0.420	1.000
119	YA	Prosody	0.3	0.8	1	0.8	1	0.933	-12.260	0.127	0.977
119	YA	Semantics	0.3	0.5	0.9	0.7	1	0.867	-9.877	0.479	0.949
120	YA	Prosody	0.2	0.4	0.7	0.9	0.8	0.850	-8.019	0.076	0.992
120	YA	Semantics	0.2	0.3	0.5	0.7	0.9	0.900	-3.807	0.053	0.996
121	YA	Prosody	0.3	0.4	0.9	0.9	0.8	0.867	-9.584	0.420	0.988
121	YA	Semantics	0.3	0.2	0.7	0.9	0.9	0.900	-5.336	0.420	0.994
122	YA	Prosody	0.4	0.7	0.9	1	1	1.000	-11.454	0.068	0.998
122	YA	Semantics	0.2	0.2	0.8	0.9	0.9	0.900	-5.747	0.420	1.000
123	YA	Prosody	0.3	0.8	0.9	0.9	1	0.936	-12.211	0.121	0.995
123	YA	Semantics	0.2	0.6	0.8	1	1	0.995	-9.223	0.076	0.990
124	YA	Prosody	0.5	0.8	0.7	0.9	0.8	0.800	-15.000	0.351	0.970
124	YA	Semantics	0.2	0.5	0.8	0.8	0.8	0.800	-10.000	0.345	1.000
125	YA	Prosody	0.4	0.8	0.8	0.9	0.9	0.870	-13.530	0.098	0.993
125	YA	Semantics	0.3	0.8	0.8	1	0.9	0.904	-12.434	0.120	0.982
126	YA	Prosody	0.4	0.8	0.9	1	1	0.981	-12.539	0.086	0.996
126	YA	Semantics	0.2	0.6	0.9	0.8	1	0.900	-10.126	0.400	0.983
127	YA	Prosody	0.6	0.6	1	0.8	1	0.945	-13.115	0.051	0.916
127	YA	Semantics	0.2	0.5	0.8	0.9	1	0.963	-8.506	0.076	0.995
201	OA	Prosody	0.3	0.3	0.7	0.8	0.4	0.633	-9.494	0.359	0.830
201	OA	Semantics	0.1	0.2	0.5	0.6	0.8	0.800	-3.627	0.049	0.980
202	OA	Prosody	0.3	0.4	0.8	0.6	0.8	0.733	-9.732	0.360	0.951
202	OA	Semantics	0.2	0.5	0.8	0.8	0.7	0.767	-10.046	0.360	0.991
203	OA	Prosody	0.3	0.2	0.6	0.8	0.7	0.750	-5.320	0.360	0.983
203	OA	Semantics	0.3	0.4	0.6	1	1	1.000	-5.209	0.080	0.989
204	OA	Prosody	0.4	0.7	0.7	0.5	0.9	0.700	-14.819	0.280	0.863
204	OA	Semantics	0.2	0.1	0.5	0.9	1	0.997	-3.967	0.107	0.999
205	OA	Prosody	0.3	0.6	0.7	0.9	0.8	0.834	-10.876	0.058	0.983
205	OA	Semantics	0.3	0.2	0.6	0.9	1	0.961	-4.976	0.135	0.991
206	OA	Prosody	0.2	0.5	0.8	0.8	0.8	0.800	-10.000	0.354	1.000
206	OA	Semantics	0.2	0.5	0.8	0.5	1	0.767	-10.035	0.472	0.868
207	OA	Prosody	0.5	0.5	1	0.9	0.9	0.933	-9.678	0.480	0.946
207	OA	Semantics	0.2	0.5	0.8	1	0.9	0.945	-8.839	0.085	0.992
208	OA	Prosody	0.3	0.5	0.9	0.8	0.9	0.867	-9.859	0.420	0.988
208	OA	Semantics	0.4	0.3	0.9	0.7	0.9	0.833	-8.155	0.420	0.949
209	OA	Prosody	0.2	0.7	0.9	0.9	0.9	0.900	-10.386	0.416	1.000
209	OA	Semantics	0.2	0.5	0.7	0.4	0.8	0.633	-10.248	0.354	0.851

210	OA	Prosody	0.3	0.5	0.8	0.8	0.9	0.870	-9.700	0.062	0.994
210	OA	Semantics	0.2	0.2	0.6	0.9	1.0	0.970	-5.033	0.120	0.998
211	OA	Prosody	0.2	0.4	0.3	0.5	0.6	0.600	-4.482	0.022	0.912
211	OA	Semantics	0.2	0.2	0.5	0.8	0.9	0.889	-4.327	0.089	0.998
212	OA	Prosody	0.2	0.5	1.0	0.9	1.0	0.967	-9.824	0.480	0.995
212	OA	Semantics	0.2	0.4	0.7	0.8	0.8	0.800	-8.470	0.074	0.999
213	OA	Prosody	0.4	0.2	0.7	0.9	0.7	0.800	-5.466	0.420	0.944
213	OA	Semantics	0.2	0.2	0.7	0.8	0.6	0.700	-7.465	0.360	0.974
214	OA	Prosody	0.4	0.4	0.8	0.6	0.7	0.700	-9.694	0.359	0.916
214	OA	Semantics	0.2	0.2	0.7	0.7	0.9	0.800	-6.217	0.198	0.980
215	OA	Prosody	0.2	0.4	0.9	0.9	1.0	0.952	-8.580	0.135	0.997
215	OA	Semantics	0.2	0.2	0.5	0.8	0.6	0.700	-5.141	0.360	0.969
216	OA	Prosody	0.4	0.3	0.4	0.7	0.6	0.650	-4.732	0.300	0.925
216	OA	Semantics	0.2	0.2	0.2	0.8	0.7	0.750	-3.031	0.360	0.994
217	OA	Prosody	0.1	0.3	0.4	0.6	0.5	0.552	-6.715	0.035	0.964
217	OA	Semantics	0.2	0.2	0.4	0.5	0.9	0.900	-0.406	0.063	0.974
218	OA	Prosody	0.2	0.3	0.5	0.5	0.6	0.558	-8.047	0.043	0.984
218	OA	Semantics	0.2	0.3	0.5	0.9	0.9	0.900	-4.542	0.112	0.995
219	OA	Prosody	0.2	0.6	0.8	0.8	0.9	0.835	-10.576	0.143	0.993
219	OA	Semantics	0.2	0.1	0.6	0.5	0.7	0.600	-7.466	0.300	0.961
220	OA	Prosody	0.4	0.3	0.7	0.6	0.4	0.567	-8.312	0.300	0.784
220	OA	Semantics	0.4	0.2	0.4	0.5	0.6	0.600	-2.921	0.033	0.873
221	OA	Prosody	0.3	0.5	0.8	0.9	0.9	0.900	-9.414	0.067	0.999
221	OA	Semantics	0.3	0.3	0.6	0.8	0.8	0.800	-6.042	0.079	0.993
222	OA	Prosody	0.2	0.4	0.8	1.0	1.0	1.000	-7.500	0.094	0.999
222	OA	Semantics	0.3	0.3	0.5	0.9	0.7	0.800	-4.920	0.419	0.963

Appendix C. Results of the study: individual recognition rates at different SNRs, individual fitted parameters of the psychometric functions and quality of fit (r; correlation between actual data and values predicted by the function). YA= Young Adult; OA=Older Adult.

Appendix D.

		Fitted Parameters															
**Age Group**	**Speech Channel**	***L* (max asymptote)**				**x_0 (threshold)**				***k* (slope)**				**r (quality of fit)**			
		**Mean**	**SD**	**Min**	**Max**	**Mean**	**SD**	**Min**	**Max**	**Mean**	**SD**	**Min**	**Max**	**Mean**	**SD**	**Min**	**Max**
**Young Adults**	**Prosody**	0.876	0.077	0.646	1	-11.403	2.385	-15.078	-4.960	0.199	0.165	0.041	0.480	0.972	0.028	0.894	0.998
	**Semantics**	0.936	0.063	0.800	1	-7.747	2.147	-12.434	-3.807	0.184	0.160	0.042	0.480	0.984	0.022	0.917	1
**Older Adults**	**Prosody**	0.777	0.138	0.552	1	-8.782	2.328	-14.819	-4.482	0.252	0.158	0.022	0.480	0.952	0.059	0.784	1
	**Semantics**	0.814	0.119	0.600	1	-5.958	2.546	-10.248	-0.406	0.211	0.146	0.033	0.472	0.967	0.043	0.851	0.999

Appendix D. Means, Standard Deviations, Minimum and Maximum values of psychometric functions' fitted parameters, averaged across participants in different age groups (young and older adults) and speech channels (prosody and semantics).

Appendix E.

Psychometric Function's Parameters- only r>0.9

	Threshold	Max recognition	Slope
Age Group	$F(1,45.71) = 17.19$, $p < .001$	$F(1,45.04) = 19.72$, $p < .001$	$F(1,46.40) = .519$, $p = .475$
Speech Channel	$F(1,43.24) = 82.14$, $p < .001$	$F(1,45.24) = 4.90$, $p = .032$	$F(1,44.99) = .821$, $p = .37$
Age Group* Speech Channel	$F(1,43.24) = 2.11$, $p = .153$	$F(1,45.24) = .185$, $p = .669$	$F(1,44.99) = .095$, $p = .759$
Model Summary	BIC = 402.19	BIC = -135.5	BIC = -47.36

Appendix E. Model Summary and results of MLM analyses for individual psychometric functions' parameters (left column: Thresholds, x_0 parameter; middle column: Max recognition, L parameter, maximum asymptote; right column: Slope, k parameter) after excluding all functions with fit quality under 0.9 (seven functions excluded, 7% of data). Significant effects are shaded.
